# Glycemic Control, Basal/Bolus Distribution, BMI and Meal Management in Children and Adolescents with Type 1 Diabetes and Advanced Hybrid Closed Loop

**DOI:** 10.3390/nu15234875

**Published:** 2023-11-22

**Authors:** Barbara Piccini, Matteo Felicioni, Benedetta Pessina, Mattia Bertini, Emilio Casalini, Chiara Ceccotti, Silvia Farina, Marta Ferrari, Lorenzo Lenzi, Francesca Monzali, Sonia Toni

**Affiliations:** 1 Endocrinology and Diabetology Unit, Meyer University Children’s Hospital IRCCS, 50139 Florence, Italy; silvia.farina@meyer.it (S.F.); marta.ferrari@meyer.it (M.F.); lorenzo.lenzi@meyer.it (L.L.); sonia.toni@meyer.it (S.T.); 2 Department of Pediatrics, University of Perugia, 06156 Perugia, Italy; felicionimatteo92@gmail.com; 3 Department of Pediatrics, Meyer University Children’s Hospital IRCCS, University of Florence, 50139 Florence, Italy; benedetta.pessina@unifi.it; 4 Department of Pediatrics, Santa Maria Alle Scotte University Hospital, University of Siena, 53100 Siena, Italy; mattia.bertini.92@gmail.com (M.B.); ceccottichiara1@gmail.com (C.C.); 5 Department of Pediatrics, Istituto Giannina Gaslini, University of Genova, 16147 Genova, Italy; emiliocasalini1@gmail.com; 6 Dietology Unit, Meyer University Children’s Hospital IRCCS, 50139 Florence, Italy; francesca.monzali@meyer.it

**Keywords:** pediatric diabetes, carbohydrate, advanced hybrid closed loop, body mass index, glycemic control

## Abstract

Evidence about the impact of advanced hybrid closed loop (AHCL) on body mass index (BMI) and eating habits in children with type 1 diabetes (T1D) is lacking. This real-world study aimed at evaluating glycemic control, BMI, meals and basal/bolus distribution in young subjects with T1D treated by AHCL. Glycemic metrics, HbA1c, basal/bolus distribution, meals/day, BMI, total daily dose (TDD), and carbohydrates/kg (CHO/kg) have been evaluated in 83 subjects, aged 13 ± 4.5 years, in manual mode, 3 and 6 months after auto-mode. Time in range (TIR) increased after 3 months, exceeding the target of 70% and was maintained at 6 months. While coefficient of variation (CV) did not change, the glucose management indicator (GMI) decreased in auto-mode (6.7 ± 0.3 vs. 7.1 ± 0.5%; *p* < 0.001), as well as HbA1c. Basal proportion decreased in favor of boluses (38.3 ± 7.3 vs. 43.6 ± 10.9%; *p* < 0.001). Meals increased at 3 and 6 months (4.4 ± 1.2 vs. 5.0 ± 1.5, *p* 0.002 and 5.1 ± 1.7, *p* < 0.001), as well as TDD/kg, without changes in BMI and CHO consumed. No differences in meal composition have arisen from food diaries. In conclusion, AHCL ensured the achievement and maintenance of target TIR in young T1D subjects. The number of meals, TDD, and insulin bolus proportion increased over time, but BMI remained stable.

## 1. Introduction

Automated insulin delivery systems (AIDs) include continuous glucose monitoring (CGM), which dialogues with an algorithm tuning insulin delivery from the pump in order to guarantee insulin therapy driven by glucose levels and their variations. All the AIDs currently available are hybrid, and they need patients entering carbohydrates (CHO) for meals. In previous real-world studies from our diabetology unit [1] and other centers [2], the use of the MiniMed^TM^ 780G system resulted in a rapid and sustained improvement in glycemic control in young T1D subjects, reaching the recommended time in range (TIR) and target HbA1c. Metabolic outcomes improved across different age groups, bridging the gap in glycemic control between younger patients and adolescents [3]. Stricter system settings (active insulin time (AIT) of 2 h and glucose target of 100 mg/dL) were associated with better glycemic control, without an increase in severe hypoglycemia [1,4]. An adequate number of boluses, by manually entering carbohydrates for each meal or snack, is crucial to achieve satisfactory glycemic control, up to the threshold of six times per day, above which no significant TIR improvement is observed [1,4]. Bolusing several times per day due to the higher flexibility, handling and freedom attained, and the possibility to easily administer insulin extra doses for snacking with continuous subcutaneous insulin infusion (CSII) could be attributed to unhealthy nutritional choices, and weight gain has been reported as a side effect of intensified insulin therapy in pediatric T1D patients. In the Diabetes Control and Complications Trial (DCCT), patients on intensive insulin therapy gained more weight over 5 years than patients in the study’s conventional arm due to fewer calories lost with glycosuria [5].

On the other hand, it is known that CSII is associated with a reduction in total daily insulin (TDD), which can positively affect body weight, as well as the reduction in hypoglycemia and this is especially true in the era of AIDs. The mean prevalence of obesity and overweight is increasing, up to 30% in youths with T1D, and obesity represents an additional risk factor for macro- and microvascular complications. It could be speculated that, because the AIDs could address and cover up errors in CHO count, some individuals could let the portion sizes increase without counting CHO accurately, or choose more fatty foods, which are difficult to manage with conventional therapy, and rely on auto-corrective boluses. There are few data available about the influence of advanced hybrid closed loop systems (AHCLs) on body weight to our knowledge, and this is especially true in children with T1D.

The aim of this study was to evaluate long-term glycemic control, body mass index (BMI) changes, meals and basal/bolus distribution in children and adolescents with T1D switching to AHCL in a real-world clinical setting.

## 2. Materials and Methods

### 2.1. Data Sources and Subjects

This retrospective observational single-center analysis included 83 pediatric T1D subjects switching to the MiniMed^TM^ 780G system and followed at the Diabetology Unit of Meyer University Children’s Hospital IRCCS in Florence, Italy. Carelink reports were collected. All patients gave written informed consent for their data to be aggregated and analyzed, and the study was approved by the local research ethical committee.

### 2.2. Outcome Variables

The percentage of TIR (70–180 mg/dL), the percentage of patients with TIR > 70% and TIR > 80%, the coefficient of variation (CV), glucose management indicator (GMI), basal/bolus distribution (daily basal insulin %, daily bolus insulin %, and daily auto-correction bolus insulin %), and HbA1c at baseline and at 3 and 6 months from the switch to MiniMed^TM^ 780G were evaluated. Moreover, the following anthropometric and nutrition parameters were considered: the number of meals/day, weight (kg), BMI (kg/m^²^), BMI z-score, TDD and TDD/kg (units), and CHO and CHO/kg (gr).

The above-mentioned outcome variables were compared across the following time periods: manual mode period (from baseline to auto-mode activation), 3 months in auto-mode (A3M, first 14 days after 3 months of auto-mode), 6 months in auto-mode (A6M, first 14 days after 6 months in auto-mode).

Only subjects with complete device follow-up were included in the respective group. The percentage of sensor use was not included, but percentage of auto-mode was considered as an indirect indicator of sensor utilization.

In our diabetology unit, the duration of the initial manual mode is not standardized and a “patient tailored” approach is adopted. The auto-mode is started when the multidisciplinary diabetes team has assessed sufficient confidence and appropriate device utilization in manual mode.

The following nutrition parameters from diaries before and after use of MiniMed^TM^ 780G were evaluated as well: Kcal, protein (gr and %), lipids (gr and %), saturated fatty acids (gr and %), CHO (gr and %), simple CHO (gr and %), and fiber (gr).

To evaluate the potential role of confounding factors, TIR, GMI, glycemic variability index, basal/bolus distribution, TDD/kg, CHO/kg, and BMI and BMI z-score were also analyzed and adjusted for gender, age (continuous or in classes ≤13 versus >13 years) and type of therapy before MiniMed^TM^ 780G (multiple daily injections (MDI) versus CSII).

### 2.3. Statistical Analysis

Descriptive statistics were used to summarize all results. These include mean and standard deviation, minimum, maximum and median with interquartile range (IQR) for continuous variables and counts and percentages for categorical variables. Summary statistics were reported with a maximum of 2 decimals, as appropriate. Continuous variables were compared between groups using Wilcoxon’s test. Categorical variables were compared between groups using the Chi-square test or Fisher’s exact test, as appropriate. Continuous and categorical variables were also plotted using boxplots and bar charts, respectively. In the box plot charts, boxes indicate the IQR, with the mid-line and the diamond representing the median and the mean, respectively. Whiskers extend from each box to the farthest point within ±1.5 × IQR. Observations outside this range are considered outliers and are denoted by circles. Mean changes for each pairwise period comparison, whether unadjusted or adjusted by the potential confounding factors, were estimated using linear mixed models to account for the within-patient correlation. Estimates along with their 95% Confidence Intervals (CIs) were provided. A pre–post comparison of parameters from diaries were performed using the exact Wilcoxon’s test. All statistical tests were based on a two-sided significance level of 0.05. All *p*-values were rounded to 3 decimal digits, and values below 0.001 were reported as “<0.001”.

SAS software, version 9.4, (SAS Institute Inc., Cary, NC, USA) was used to perform statistical analyses.

## 3. Results

### 3.1. Population Set

The study included 83 pediatric T1D individuals (38 males) who started using the MiniMed^TM^ 780G. The mean age was 13 ± 4.5 years, and the mean age at diagnosis was 6 ± 3.8 years, with a mean diabetes duration of 7 ± 4.5 years. Before AHCL, 37% of patients were on MDI therapy, and 63% were on CSII. No patients dropped out of the AHCL. Table 1 outlines baseline demographic data for the whole study cohort.

### 3.2. Device Settings

The mean duration of the run-in period before switching to auto-mode was 32.6 ± 38.6 days. The mean percentage of time spent in auto-mode was stable during the follow-up and was 95.7 ± 8.8% at A3M and 92.9 ± 11.1% at A6M.

### 3.3. TIR

**Figure 1:**
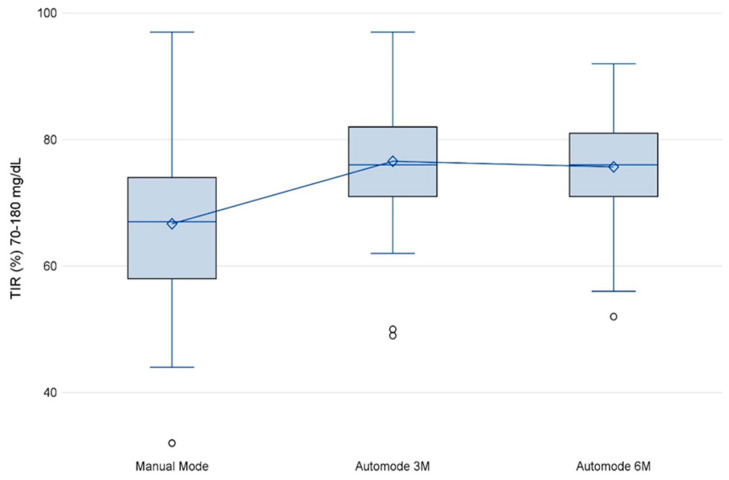
Percentage of TIR by time-period. Abbreviations: Auto-mode 3M, first 14 days after 3 months; Auto-mode 6M, first 14 days after 6 months; TIR, time in range.

TIR showed a statistically significant improvement between manual and auto-mode at all follow-up periods (A3M and A6M) (Figure 1). The mean TIR in manual mode was below the therapeutic target (66.7 ± 13.2%), reaching 76.6 ± 8.5% at A3M (*p* < 0.001), and 75.7 ± 7.8% at A6M (*p* < 0.001).

During the manual mode period, 35% of subjects showed TIR > 70%, and 15% had TIR > 80%. The percentage of patients with TIR > 70% significantly increased to 77% (*p* 0.001) and 80% (*p* < 0.001), respectively, at A3M and at A6M. The percentage of patients with TIR > 80% increased to 33% (*p* 0.13) at A3M and 26% (*p* 0.31) at A6M.

No severe hypoglycemia or DKA episodes occurred during the study.

### 3.4. Glycemic Variability and GMI

Sensor glucose CV remained fairly constant during the whole follow-up, while GMI reported a significant decrease after switching to auto mode (7.1 ± 0.5% in manual mode vs. 6.7 ± 0.3% at A3M and 6.7 ± 0.5% at A6M) (Table 2).

### 3.5. Basal/Bolus Insulin Changes

A statistically significant difference of basal and bolus volumes (%) was observed between the manual and auto-mode period. Basal insulin decreased from 43.6 ± 10.9% in manual mode to 38.3 ± 7.3% and 39.2 ± 7.7% at A3M and A6M, respectively (*p* < 0.001), while bolus insulin increased from 56.4 ± 10.9% to 61.3 ± 8% and 60.8 ± 7.7% at A3M and A6M, respectively (*p* < 0.001). Auto-correction bolus volume was 24.1 ± 9.9% at A3M and remained constant during follow-up (Supplementary Table S1).

### 3.6. HbA1c

A significant decrease in HbA1c (%) to recommended values was observed when switching to MiniMed^TM^ 780G from 7.2 ± 0.7% to 6.9 ± 0.7% (*p* 0.002) after 3 months and to 6.9 ± 0.6% (*p* 0.003) after 6 months.

### 3.7. Anthropometric and Nutrition Parameters

The daily number of meals significantly increased with the switch to auto-mode and the TDD/kg increased as well, with stability of BMI z-score and CHO intake across time (Table 3).

### 3.8. Multivariate Analysis

#### 3.8.1. Gender and Age

**Figure 2:**
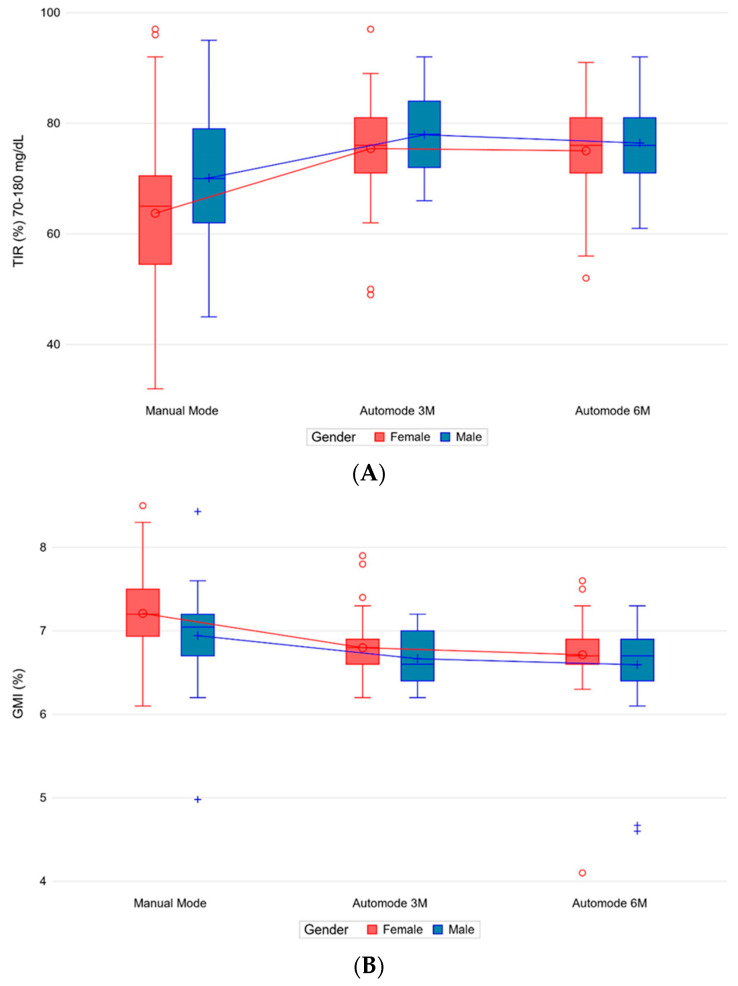
Percentages of TIR (**A**) and GMI (**B**) by time period and gender. Abbreviations: Auto-mode 3M, first 14 days after 3 months; Auto-mode 6M, first 14 days after 6 months; GMI, glucose management indicator; TIR, time in range.

In the multivariate model, when controlling for gender and age (≤13 years vs. >13 years) as potential outcome predictors together with the time period, mean changes across time periods for all outcomes remained virtually unchanged. Both variables were statistically associated with TIR and GMI. Male patients seemed to fare significantly better than female patients in terms of TIR (*p* 0.037) and GMI (*p* 0.016), regardless of the time period (Figure 2A,B). No statistically significant difference was found in CV, basal/bolus distribution as well as TDD/kg, CHO/kg, BMI, and BMI z-score between females and males (Supplementary Table S2).

**Figure 3:**
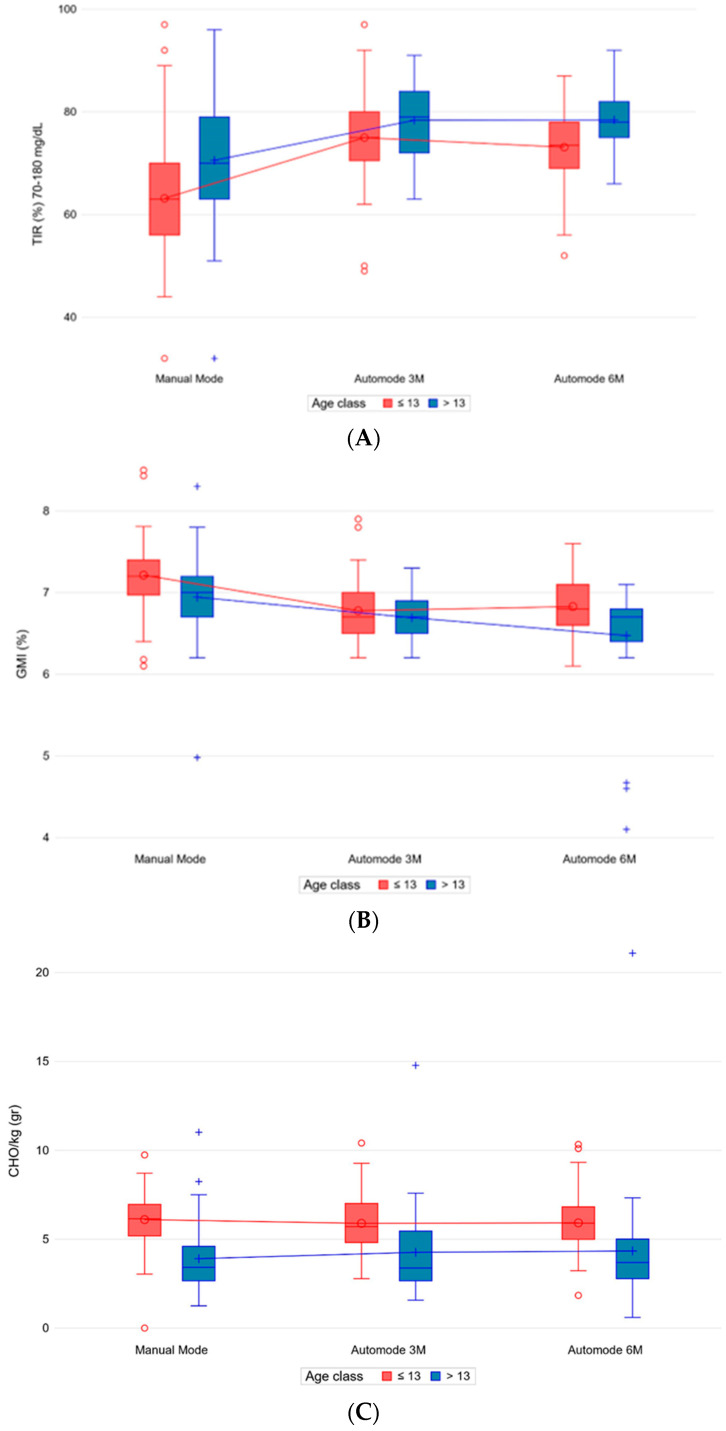
Percentages of TIR (**A**) and GMI (**B**) by time period and age, CHO/kg (**C**) by time period and age. Abbreviations: Auto-mode 3M, first 14 days after 3 months; Auto-mode 6M, first 14 days after 6 months; GMI, glucose management indicator; TIR, time in range.

Older patients showed better TIR (*p* 0.003) and GMI (*p* 0.003) (Figure 3A,B), without a difference in CV, basal/bolus distribution and TDD/kg (Supplementary Table S3).

Moreover, older age was statistically associated with less CHO/kg consumed (*p* 0.001), without a statistically significant difference in BMI z-score (Figure 3C).

#### 3.8.2. Previous Therapy

Previous T1D treatment was included in a multivariate model together with the time period, but no significant changes in outcomes were reported for any time period, with the exception of basal/bolus distribution, which showed an increased basal proportion and a decreased bolus proportion in subjects previously treated by CSII (Supplementary Table S4).

### 3.9. Nutrition Parameters from Diaries before and after Use of MiniMed^TM^ 780G

The before/after AHCL food diary of a subgroup of subjects showed no differences in meal composition or calories intake (Table 4).

## 4. Discussion

In the study cohort, AHCL therapy has enabled the achievement and maintenance of the recommended goal of TIR > 70%, and in one-third of the patients, the optimal outcome of TIR > 80% was achieved. GMI was significantly reduced, while no changes in CV were observed, but it was already below the recommended target (<36%) before auto-mode initiation [6]. The improvement of glycemic metrics was mirrored by HbA1c, which was above the optimal target before AHCL initiation and reached median levels of 6.9% at 3 and 6 months. These findings are consistent with both randomized controlled trials and real-world studies that have shown that the MiniMed^TM^ 780G system safely achieves recommended glycemic targets in most adolescents and adults with T1D [1,7,8]. Interestingly, in our study, BMI z-score and CHO intake remained stable throughout all the follow-up duration, eliminating concerns that the use and spread of this automated technology could lead to a deterioration of knowledge of the exact carbohydrate count and to less healthy eating choices and more frequent snacking, resulting in weight gain and possibly obesity. Studies that observed BMI in children with T1D using insulin pumps before the new era of AHCL showed results that were not conclusive [9]. Alderisio et al. observed body weight trajectories, in the long-term (6–10 years), of a cohort of adult T1D patients on CSII or MDI, and they showed comparable body weight gain (0.5 kg per year) despite improved glycemic control and decreased insulin doses with CSII [10]. Studies on the association between AHCL and both BMI changes and dietary habits among young subjects with T1D are scarce. In line with what we observed, Seget et al. reported that during a 1-year follow-up of 50 children and adolescents with AHCL, BMI z-scores did not change significantly [11]. On the other hand, Lawton et al. explored the impact of AHCL use on food choices among 24 T1D people in the APCam11 trial with interviews before and after three months of closed loop, and some participants reported increased snacking and portion sizes and the consumption of fatty, high calorie foods [12]. In another 3-month trial with HCL (Medtronic MiniMed^TM^ 670G), weight gains of 1.4 and 1 kg were also observed among adult (*n* = 94) and adolescent (*n* = 30) users, respectively [13]. Our data over a longer period and in a real-world setting showed that the number of meals increased at 3 and 6 months after the switch to AHCL, as well as the TDD/kg, but notably BMI z-score and CHO intake remained stable. On the contrary, Messer et al. [14] described that the number of meal boluses remained unchanged with a decreasing trend between baseline and 6 months (4.4 versus 4) in 191 youths with T1D who started AHCL with Tandem t:slim X2 with Control-IQ, but with a lower TIR at 6 months than in our cohort (TIR 66%). In our study cohort the percentage of basal insulin reduced in favor of boluses. Auto-correction bolus volume was consistently 1/5 of total bolus insulin during follow-up, comparable to previous studies [8]. It could be speculated that the increased number of user-initiated meal boluses detected in our cohort, together with the constant intervention of auto-correction boluses during the follow-up, means that children and adolescents switched to the AHCL system were more prone to bolus for every meal or snack, without increasing the overall CHO intake, and this an essential indicator of self-management behavior, strongly associated with glycemic control. Remarkably, in a subgroup of patients who filled out a food diary before and after AHCL, no differences in meal composition or calories intake were highlighted, excluding qualitative food choices worsening. Moreover, we found that TIR and GMI were better in subjects aged > 13 years, with lower CHO/kg daily intake compared to younger children. This could be explained either by the fact that adolescents do not need five meals a day and snack less frequently or that they omit CHO input, but this explanation is less likely considering their optimal glycemic control without change in BMI z-score. In our cohort, male children showed a better TIR than females, which is in line with what was evaluated by Castaneda et al., who found that male users had on average 0.9% higher TIR, but without a different amount of CHO/kg consumed and BMI z-score change between females and males [4]. These findings could be due to the sample size and to potential selection bias in the real-world setting and need to be confirmed with a larger cohort of patients.

Looking at treatment modality before the switch to AHCL, patients naive to advanced technology, such as MDI patients, did not show any difference in glycemic control and BMI z-score change. Subjects previously treated by CSII showed an increased basal proportion and a decreased bolus proportion as if the automation does the job, requiring fewer correction boluses and interventions from the subjects.

In our clinical practice, T1D candidates for starting AHCL therapy and their family follow a learning plan, which includes different members of the diabetologic team, such as a pediatric diabetologist, diabetological nurse, dietician and psychologist. A training course for CHO counting with the dietician and the subsequent verification of the acquisition of the necessary skills is a “conditio sine qua non” for AIDs use, considering that the insulin/CHO ratio is a modifiable factor together with the timing of bolusing and other settings such as AIT and glucose target for the Medtronic MiniMed^TM^ 780G. Petrovski et al. have shown that a simplified meal announcement with a preset of different fixed-carbohydrate amounts could be used, with 67% of patients in the fix group reaching the target TIR, even with a 6.8% TIR difference in favor of the precise CHO count [15]. Bolus omission and bolus delay are very common, especially among adolescents with T1D, and even though Tornese et al. observed that unannounced CHO snack of <20 g of CHO resulted in a tolerable glucose excursion in children and adolescents treated by the Medtronic MiniMed^TM^ 780G [16], it is of utmost importance to ensure patients and their families receive retraining in CHO counting periodically, as well as verifying that meals are announced, and if the insulin/CHO settings are still reliable or if it is the algorithm that does the job. Bolus omission seems an issue that does not concern the study cohort, and the number of meals increased over time. The educational role of the pediatric diabetologist, as well as the patient-centered, tailored medical support, are key to a win–win solution, even with the most advanced technologies. The duration of the manual mode is not established a priori, and indeed, it was quite variable in our cohort of patients due to the fact that some requirements have to be met before switching to the auto-mode, and some subjects who use it need time to trust the system and to use it properly. Interestingly, no dropouts were observed in the large study cohort, even if the discontinuation of AID devices has been reported at up to 30% in youths, mostly within the first 3 months of use [17]. This could be due to the results achieved in terms of glycemic control and to the high level of patient involvement adjusting the smart-guard settings and monitoring glycemic metrics with telemedicine visits every two weeks for the first quarter.

The current study has inherent strengths and limitations. Given the retrospective design, selection bias could be possible. No information about physical activity or metabolic control before AHCL was collected and precise dietary evaluation before and after the switch to AHCL was available only for a subgroup of patients in order to evaluate changes in eating habits. Even though the percentage of sensor use was not analyzed, the mean percentage of time in auto-mode was high in our cohort (>90% at all time points) and stable through follow-up. A prolonged follow-up period could be necessary in order to confirm these results. Points of strength of this study are the real-world data, which provide the opportunity to assess clinical outcomes in real-life practice and the multivariate nature of the analysis, including demographic and anthropometric characteristics.

In conclusion, this study showed how the AHCL MiniMed^TM^ 780G provides both the achievement of good glycemic control and the maintenance of a stable BMI z-score and CHO intake over time, despite the increase in the meals per day and TDD, in a population of children and adolescents with T1D.

## Figures and Tables

**Table 1:** Baseline and demographic data.

Baseline Characteristics	Summary Statistics	Total (*n* = 83)
Age (yrs)	*n*	83 (100.0%)
	Mean ± SD	13.0 ± 4.5
	Median (IQR)	12.5 (9.3–15.8)
	Min–Max	3.3–22.3
Gender (male)	%, *n*/Pts	45.8% (38/83)
Age at diagnosis (yrs)	*n*	83 (100.0%)
	Mean ± SD	6.0 ± 3.8
	Median (IQR)	5.7 (2.3–8.5)
	Min–Max	0.9–16.6
DM duration (yrs)	*n*	83 (100.0%)
	Mean ± SD	7.0 ± 4.5
	Median (IQR)	6.6 (2.8–9.4)
	Min–Max	0.3–19.2
Previous therapy		
MDI	%, *n*/Pts	37.3% (31/83)
CSII	%, *n*/Pts	62.7% (52/83)
Manual mode duration (days)	*n*	83 (100.0%)
	Mean ± SD	32.6 ± 38.6
	Median (IQR)	20.0 (13.0–32.0)
	Min–Max	3.0–232.0

Abbreviations: CSII, continuous subcutaneous insulin infusion; DM, diabetes mellitus; IQR, interquartile range; MDI, multiple daily injections; *n*, number; Pts, patients; SD, standard deviation; yrs, years.

**Table 2:** Glycemic variability index and GMI—mean change across different time points (95% CI).

	Summary Statistics	Manual Mode (*n* = 83)	Auto-Mode 3M (*n* = 83)	Auto-Mode 6M (*n* = 83)	Mean Change 95% CI *p*-Value	
					Auto-Mode 3M (vs. Manual Mode)	Auto-Mode 6M (vs. Manual Mode)
SG CV (%)	*n*	82 (98.8%)	83 (100.0%)	81 (97.6%)	−0.9 (−2.4–0.5) 0.204	−0.2 (−1.4–1.0) 0.754
	Mean ± SD	34.2 ± 6.2	33.3 ± 4.5	34.1 ± 5.0		
	Median (IQR)	32.7 (31.2–38.1)	33.0 (30.3–36.2)	33.9 (30.5–37.0)		
	Min–Max	21.3–66.7	22.0–44.4	24.1–50.0		
GMI (%)	*n*	82 (98.8%)	83 (100.0%)	81 (97.6%)	−0.3 (−0.5–0.2) **<0.001**	−0.4 (−0.5–0.3) **<0.001**
	Mean ± SD	7.1 ± 0.5	6.7 ± 0.3	6.7 ± 0.5		
	Median (IQR)	7.1 (6.9–7.4)	6.7 (6.5–7.0)	6.7 (6.5–6.9)		
	Min–Max	5.0–8.5	6.2–7.9	4.1–7.6		

Abbreviations: Auto-mode 3M, first 14 days after 3 months; Auto-mode 6M, first 14 days after 6 months; CI, confidence interval; GMI, glucose management indicator; IQR, interquartile range; SD, standard deviation; SG CV, sensor glucose coefficient of variation. Bold indicates statistically significant.

**Table 3:** Anthropometric and nutrition parameters—mean change across different time points (95% CI).

	Summary Statistics	Manual Mode (*n* = 83)	Auto-Mode 3M (*n* = 83)	Auto-Mode 6M (*n* = 83)	Mean Change 95% CI *p*-Value	
					Auto-Mode 3M (vs. Manual Mode)	Auto-Mode 6M (vs. Manual Mode)
Number of meals/day	*n*	82 (98.8%)	83 (100.0%)	80 (96.4%)	0.6 (0.2–1.0) **0.002**	0.7 (0.4–1.0) **<0.001**
	Mean ± SD	4.4 ± 1.2	5.0 ± 1.5	5.1 ± 1.7		
	Median (IQR)	4.3 (3.7–5.0)	4.8 (4.0–5.6)	4.9 (4.0–5.9)		
	Min–Max	1.3–7.5	2.4–8.7	1.8–10.7		
Weight (Kg)	*n*	83 (100.0%)	83 (100.0%)	81 (97.6%)	0.7 (−0.0–1.4) 0.052	1.7 (1.2–2.2) **<0.001**
	Mean ± SD	43.2 ± 18.2	43.9 ± 18.0	45.1 ± 18.2		
	Median (IQR)	37.5 (30.0–60.0)	38.5 (31.1–60.0)	40.7 (31.3–60.0)		
	Min–Max	14.9–84.0	14.7–84.9	15.1–88.5		
BMI (kg/m²)	*n*	83 (100.0%)	82 (98.8%)	81 (97.6%)	0.0 (−0.2–0.3) 0.775	0.1 (−0.1–0.3) 0.205
	Mean ± SD	19.0 ± 3.2	19.0 ± 3.1	19.2 ± 3.2		
	Median (IQR)	18.3 (16.5–21.3)	18.2 (16.6–20.7)	18.9 (16.5–21.2)		
	Min–Max	14.3–27.0	13.7–27.2	14.1–28.7		
BMI z-score	*n*	83 (100.0%)	82 (98.8%)	80 (96.4%)	−0.1 (−0.2–0.0) 0.154	−0.1 (−0.2–0.0) 0.050
	Mean ± SD	0.2 ± 0.9	0.1 ± 0.9	0.1 ± 0.9		
	Median (IQR)	0.2 (−0.3–0.9)	0.2 (−0.4–0.8)	0.2 (−0.3–0.8)		
	Min–Max	−2.1–1.9	−2.1–1.8	−2.1–1.9		
TDD (units)	*n*	82 (98.8%)	83 (100.0%)	81 (97.6%)	2.4 (0.6–4.3) **0.010**	4.3 (2.9–5.6) **<0.001**
	Mean ± SD	31.0 ± 16.5	33.3 ± 17.8	35.4 ± 17.7		
	Median (IQR)	27.9 (18.3–38.4)	30.6 (20.6–42.9)	32.6 (22.8–43.1)		
	Min–Max	4.8–78.8	5.7–80.5	6.0–85.1		
TDD/Kg (units)	*n*	83 (100.0%)	83 (100.0%)	81 (97.6%)	0.1 (0.0–0.1) **0.008**	0.1 (0.0–0.1) **<0.001**
	Mean ± SD	0.7 ± 0.2	0.8 ± 0.2	0.8 ± 0.2		
	Median (IQR)	0.7 (0.6–0.9)	0.7 (0.6–1.0)	0.8 (0.7–0.9)		
	Min–Max	0.0–1.2	0.2–1.4	0.1–1.4		
CHO (gr)	*n*	82 (98.8%)	83 (100.0%)	81 (97.6%)	6.3 (−13–25.6) 0.522	12.7 (−2.2–27.7) 0.094
	Mean ± SD	197.3 ± 81.7	203.5 ± 86.7	210.7 ± 104.0		
	Median (IQR)	182.0 (149.0–229.0)	184.0 (154.0–236.0)	191.0 (144.0–257.0)		
	Min–Max	64.0–605.0	81.0–509.0	31.0–814.0		
CHO/Kg (gr)	*n*	83 (100.0%)	83 (100.0%)	81 (97.6%)	0.1 (−0.4–0.5) 0.810	0.1 (−0.3–0.4) 0.666
	Mean ± SD	5.1 ± 2.1	5.1 ± 2.1	5.2 ± 2.6		
	Median (IQR)	5.1 (3.4–6.7)	5.2 (3.4–6.4)	5.0 (3.5–6.3)		
	Min–Max	0.0–11.0	1.6–14.8	0.6–21.1		

Abbreviations: Auto-mode 3M, first 14 days after 3 months; Auto-mode 6M, first 14 days after 6 months; BMI, body mass index; CI, confident interval; IQR, interquartile range; SD, standard deviation; TDD, total daily dose. Bold indicates statistically significant.

**Table 4:** Nutritional parameters.

	Summary Statistics	Pre-780G (*n* = 83)	Post-780G (*n* = 83)	*p*-Value
Kcal	*n*	8 (9.6%)	8 (9.6%)	0.461
	Mean ± SD	1622.3 ± 464.4	1538.4 ± 371.9	
	Median (IQR)	1688.8 (1513.5–1871.0)	1638.0 (1419.5–1686.8)	
	Min–Max	637.0–2195.0	771.0–2048.0	
P (gr)	*n*	8 (9.6%)	8 (9.6%)	0.195
	Mean ± SD	62.5 ± 18.1	55.0 ± 21.6	
	Median (IQR)	62.8 (56.2–71.6)	52.9 (43.0–59.9)	
	Min–Max	27.8–90.9	27.4–101.2	
P (%)	*n*	8 (9.6%)	8 (9.6%)	0.195
	Mean ± SD	15.7 ± 2.4	14.2 ± 3.5	
	Median (IQR)	14.9 (14.5–17.0)	13.6 (12.1–16.6)	
	Min–Max	12.1–20.3	9.5–19.8	
L (gr)	*n*	8 (9.6%)	8 (9.6%)	0.641
	Mean ± SD	63.6 ± 22.9	59.3 ± 19.8	
	Median (IQR)	70.4 (49.1–75.3)	63.5 (47.8–71.0)	
	Min–Max	24.6–94.5	25.5–84.9	
L (%)	*n*	8 (9.6%)	8 (9.6%)	0.844
	Mean ± SD	35.0 ± 6.1	34.2 ± 6.2	
	Median (IQR)	36.7 (32.0–39.0)	34.0 (31.2–36.8)	
	Min–Max	22.6–41.5	23.9–45.4	
SFA (gr)	*n*	8 (9.6%)	8 (9.6%)	0.109
	Mean ± SD	17.8 ± 4.5	15.4 ± 5.3	
	Median (IQR)	17.4 (15.8–20.8)	16.6 (14.5–18.4)	
	Min–Max	9.7–24.6	3.3–20.8	
SFA (%)	*n*	8 (9.6%)	8 (9.6%)	0.195
	Mean ± SD	10.3 ± 2.2	8.7 ± 2.1	
	Median (IQR)	9.5 (8.7–12.2)	9.1 (8.4–9.8)	
	Min–Max	7.5–13.7	3.9–10.9	
CHO (gr)	*n*	8 (9.6%)	8 (9.6%)	1.000
	Mean ± SD	208.8 ± 71.7	208.3 ± 46.3	
	Median (IQR)	208.8 (196.8–259.2)	207.6 (191.8–246.1)	
	Min–Max	53.0–288.2	114.8–260.7	
CHO (%)	*n*	8 (9.6%)	8 (9.6%)	0.148
	Mean ± SD	47.1 ± 8.4	51.4 ± 5.4	
	Median (IQR)	46.1 (43.5–54.8)	53.1 (47.5–55.7)	
	Min–Max	31.2–56.5	41.4–56.8	
Simple CHO (gr)	*n*	8 (9.6%)	8 (9.6%)	0.383
	Mean ± SD	48.9 ± 25.5	42.4 ± 11.6	
	Median (IQR)	44.5 (28.4–69.3)	39.7 (32.1–53.2)	
	Min–Max	17.1–89.9	29.7–59.7	
Simple CHO (%)	*n*	8 (9.6%)	8 (9.6%)	0.742
	Mean ± SD	11.3 ± 4.6	10.8 ± 3.5	
	Median (IQR)	10.8 (8.0–14.4)	10.2 (8.3–11.9)	
	Min–Max	4.9–19.3	7.3–18.2	
Fiber (gr)	*n*	8 (9.6%)	8 (9.6%)	0.195
	Mean ± SD	17.7 ± 6.2	15.2 ± 3.6	
	Median (IQR)	18.5 (14.5–21.5)	15.0 (13.0–17.4)	
	Min–Max	6.3–26.9	9.5–21.2	

Abbreviations: CHO, carbohydrates; CI, confident interval; IQR, interquartile range; SD, standard deviation; P, protein; L, lipid; SFA, saturated fatty acids.

## Data Availability

The dataset used and/or analyzed during this study is available from the corresponding author upon reasonable request.

## Supplementary Materials

The following are available online at https://www.mdpi.com/article/10.3390/nu15234875/s1, Table S1: Basal-bolus insulin changes, Table S2: Multivariate models by gender, Table S3: Multivariate models by age, Table S4: Multivariate models by previous therapy.

## References

[1] Piccini B., Pessina B., Casalini E., Lenzi L., Toni S. (2022). Long-term effectiveness of advanced hybrid closed loop in children and adolescents with type 1 diabetes. Pediatr. Diabetes.

[2] Beato-Víbora P.I., Gallego-Gamero F., Ambrojo-López A., Gil-Poch E., Martín-Romo I., Arroyo-Díez F.J. (2021). Rapid Improvement in Time in Range After the Implementation of an Advanced Hybrid Closed-Loop System in Adolescents and Adults with Type 1 Diabetes. Diabetes Technol. Ther..

[3] Arrieta A., Battelino T., Scaramuzza A.E., Da Silva J., Castañeda J., Cordero T.L., John Shin J., Cohen O. (2022). Comparison of MiniMed 780G system performance in users aged younger and older than 15 years: Evidence from 12,870 real-world users. Diabetes Obes. Metab..

[4] Castañeda J., Mathieu C., Aanstoot H., Arrieta A., Da Silva J., Shin J., Cohen O. (2022). Predictors of time in target glucose range in real-world users of the MiniMed 780G system. Diabetes Obes. Metab..

[5] Mehta S.N., Andersen H.U., Abrahamson M.J., Wolpert H.A., Hommel E.E., McMullen W., Ridderstråle M. (2017). Changes in HbA1c and weight following transition to continuous subcutaneous insulin infusion therapy in adults with type 1 diabetes. J. Diabetes Sci. Technol..

[6] Battelino T., Danne T., Bergenstal R.M., Amiel S.A., Beck R., Biester T., Bosi E., Buckingham B.A., Cefalu W.T., Close K.L. (2019). Clinical targets for continuous glucose monitoring data interpretation: Recommendations from the international consensus on time in range. Diabetes Care.

[7] Bergenstal R.M., Nimri R., Beck R.W., Criego A., Laffel L., Schatz D., Battelino T., Danne T., Weinzimer S.A., Sibayan J. (2021). A comparison of two hybrid closed-loop systems in adolescents and young adults with type 1 diabetes (FLAIR): A multicentre, randomized, crossover trial. Lancet.

[8] Da Silva J.D., Lepore G., Battelino T., Arrieta A., Castaneda J., Grossman B., Shin J., Cohen O. (2022). Real-world performance of the MiniMed™ 780G system: First report of outcomes from 4120 users. Diabetes Technol. Ther..

[9] REPOSE Study Group (2017). Relative effectiveness of insulin pump treatment over multiple daily injections and structured education during flexible intensive insulin treatment for type 1 diabetes: Cluster randomized trial (REPOSE). BMJ.

[10] Alderisio A., Bozzetto L., Franco L., Riccardi G., Rivellese A.A., Annuzzi G. (2019). Long-term body weight trajectories and metabolic control in type 1 diabetes patients on insulin pump or multiple daily injections: A 10-year retrospective controlled study. Nutr. Metab. Cardiovasc. Dis..

[11] Seget S., Jarosz-Chobot P., Ochab A., Polanska J., Rusak E., Witoszek P., Chobot A. (2022). Body mass index, basal insulin and glycemic control in children with type 1 diabetes treated with the advanced hybrid closed loop system remain stable-1-year prospective, observational, two-center study. Front. Endocrinol..

[12] Lawton J., Blackburn M., Rankin D., Allen J., Campbell F., Leelarathna L., Tauschmann M., Thabit H., Wilinska M.E., Hovorka R. (2019). The impact of using a closed-loop system on food choices and eating practices among people with Type 1 diabetes: A qualitative study involving adults, teenagers and parents. Diabet. Med..

[13] Garg S.K., Weinzimer S.A., Tamborlane W.V., Buckingham B.A., Bode B.W., Bailey T.S., Brazg R.L., Ilany J., Slover R.H., Anderson S.M. (2017). Glucose Outcomes with the In-Home Use of a Hybrid Closed-Loop Insulin Delivery System in Adolescents and Adults with Type 1 Diabetes. Diabetes Technol. Ther..

[14] Messer L.H., Berget C., Pyle L., Vigers T., Cobry E., Driscoll K.A., Forlenza G.P. (2021). Real-World Use of a New Hybrid Closed Loop Improves Glycemic Control in Youth With Type 1 Diabetes. Diabetes Technol. Ther. Diabetes.

[15] Petrovski G., Campbell J., Pasha M., Day E., Hussain K., Khalifa A., van den Heuvel T. (2023). Simplified Meal Announcement Versus Precise Carbohydrate Counting in Adolescents With Type 1 Diabetes Using the MiniMed 780G Advanced Hybrid Closed Loop System: A Randomized Controlled Trial Comparing Glucose Control. Diabetes Care.

[16] Tornese G., Carletti C., Giangreco M., Nisticò D., Faleschini E., Barbi E. (2022). Carbohydrate Tolerance Threshold for Unannounced Snacks in Children and Adolescents with Type 1 Diabetes Using an Advanced Hybrid Closed-Loop System. Diabetes Care.

[17] Berget C., Messer L.H., Vigers T., Frohnert B.I., Pyle L., Wadwa R.P., Driscoll K.-A., Forlenza G.P. (2020). Six months of hybrid closed loop in the real-world: An evaluation of children and young adults using the 670G system. Pediatr. Diabetes.

