# Supplementary material for: Glycemic Control, Basal/Bolus Distribution, BMI and Meal Management in Children and Adolescents with Type 1 Diabetes and Advanced Hybrid Closed Loop
Source: Nutrients. 2023 Nov 22;15(23):4875. doi: 10.3390/nu15234875 (PMC10707798; doi:10.3390/nu15234875)
Supplement: Supplementary file 1 [file nutrients-15-04875-s001.zip › Table 1 Supplementary (1).pdf]

Table 1 Supplementary. Basal-bolus insulin changes

	Summary statistics	Manual Mode (N = 83)	Automode 3M (N = 83)	Automode 6M (N = 83)	Mean change 95% CI p-value	
					Automode 3M (vs. Manual Mode)	Automode 6M (vs. Manual Mode)
Basal volume (%)	N	82 (98.8%)	83 (100.0%)	81 (97.6%)	-5.3 (-7.8--2.8) **<.001**	-4.3 (-6.4--2.1) **<.001**
	Mean ± SD	43.6 ± 10.9	38.3 ± 7.3	39.2 ± 7.7		
	Median (IQR)	42.5 (36.0- 50.0)	38.0 (32.0- 42.0)	39.0 (33.0- 44.0)		
	Min-Max	21.0 - 71.0	25.0 - 61.0	27.0 - 67.0		
Correction Bolus volume (%)	N	0 (0.0%)	83 (100.0%)	80 (96.4%)	-	-
	Mean ± SD	. ± .	24.1 ± 9.9	24.5 ± 10.0		
	Median (IQR)	. (. - .)	25.0 (18.0- 30.0)	24.0 (17.0- 32.0)		
	Min-Max	. - .	1.0 - 58.0	7.0 - 66.0		
Bolus volume (%) (including correction)	N	82 (98.8%)	83 (100.0%)	81 (97.6%)	4.8 (2.2-7.4) **<.001**	4.3 (2.0-6.5) **<.001**
	Mean ± SD	56.4 ± 10.9	61.3 ± 8.0	60.8 ± 7.7		
	Median (IQR)	57.5 (50.0- 64.0)	62.0 (58.0- 68.0)	61.0 (56.0- 67.0)		
	Min-Max	29.0 - 79.0	29.0 - 75.0	33.0 - 73.0		

Abbreviations: Automode 3M, first 14 days after 3 months; Automode 6M, first 14 days after 6 months; CI, confidence interval; IQR, interquartile range; SD, standard deviation. Bold indicates statistically significant.
