# Supplementary material for: Glycemic Control, Basal/Bolus Distribution, BMI and Meal Management in Children and Adolescents with Type 1 Diabetes and Advanced Hybrid Closed Loop
Source: Nutrients. 2023 Nov 22;15(23):4875. doi: 10.3390/nu15234875 (PMC10707798; doi:10.3390/nu15234875)
Supplement: Supplementary file 1 [file nutrients-15-04875-s001.zip › Table 2 Supplementary.pdf]

Table 2 Supplementary. Multivariate models by gender

Outcome	Variable	Category	Mean change (95% CI)	p-value
TIR (%) 70-180 mg/dL	Period	Automode 3M (vs. Manual Mode)	9.9 (7.3-12.5)	**<.001**
	Period	Automode 6M (vs. Manual Mode)	9.0 (6.9-11.1)	**<.001**
	Gender (Male vs. Female)		3.9 (0.2-7.5)	**0.037**
SG CV (%)	Period	Automode 3M (vs. Manual Mode)	-0.9 (-2.4-0.5)	0.205
	Period	Automode 6M (vs. Manual Mode)	-0.2 (-1.4-1.0)	0.753
	Gender (Male vs. Female)		0.1 (-1.7-1.9)	0.883
GMI (%)	Period	Automode 3M (vs. Manual Mode)	-0.3 (-0.5--0.2)	**<.001**
	Period	Automode 6M (vs. Manual Mode)	-0.4 (-0.5--0.3)	**<.001**
	Gender (Male vs. Female)		-0.2 (-0.3--0.0)	**0.016**
Basal volume (%)	Period	Automode 3M (vs. Manual Mode)	-5.3 (-7.8--2.8)	**<.001**
	Period	Automode 6M (vs. Manual Mode)	-4.3 (-6.4--2.1)	**<.001**
	Gender (Male vs. Female)		-0.3 (-3.2-2.5)	0.814
Bolus volume (%)	Period	Automode 3M (vs. Manual Mode)	4.8 (2.2-7.4)	**<.001**
	Period	Automode 6M (vs. Manual Mode)	4.3 (2.0-6.5)	**<.001**
	Gender (Male vs. Female)		0.1 (-2.8-3.0)	0.942
TDD/kg (units)	Period	Automode 3M (vs. Manual Mode)	0.1 (0.0-0.1)	**0.008**
	Period	Automode 6M (vs. Manual Mode)	0.1 (0.0-0.1)	**<.001**
	Gender (Male vs. Female)		0.0 (-0.1-0.1)	0.580
CHO/kg (gr)	Period	Automode 3M (vs. Manual Mode)	0.1 (-0.4-0.5)	0.810
	Period	Automode 6M (vs. Manual Mode)	0.1 (-0.3-0.4)	0.667
	Gender (Male vs. Female)		0.5 (-0.3-1.4)	0.235
BMI (kg/m²)	Period	Automode 3M (vs. Manual Mode)	0.0 (-0.2-0.3)	0.774
	Period	Automode 6M (vs. Manual Mode)	0.1 (-0.1-0.3)	0.205
	Gender (Male vs. Female)*		0.7 (-0.7-2.0)	0.350
BMI z score	Period	Automode 3M (vs. Manual Mode)	-0.1 (-0.2-0.0)	0.154
	Period	Automode 6M (vs. Manual Mode)	-0.1 (-0.2-0.0)	0.051
	Gender (Male vs. Female)*		-0.2 (-0.5-0.2)	0.407

*interaction test is significant

Abbreviations: Automode 3M, first 14 days after 3 months; Automode 6M, first 14 days after 6 months; BMI, body mass index; CI, confidence interval; GMI, glucose management indicator; SG CV, sensor glucose coefficient of variation; TDD, total daily dose; TIR, time in range. Bold indicates statistically significant.
