# Supplementary material for: Glycemic Control, Basal/Bolus Distribution, BMI and Meal Management in Children and Adolescents with Type 1 Diabetes and Advanced Hybrid Closed Loop
Source: Nutrients. 2023 Nov 22;15(23):4875. doi: 10.3390/nu15234875 (PMC10707798; doi:10.3390/nu15234875)
Supplement: Supplementary file 1 [file nutrients-15-04875-s001.zip › Table 4 Supplementary.pdf]

Table 4 Supplementary. Multivariate models by previous therapy

Outcome	Variable	Category	Mean change (95% CI)	p-value
TIR (%) 70-180 mg/dL	Period	Automode 3M (vs. Manual Mode)	9.9 (7.3-12.5)	**<.001**
	Period	Automode 6M (vs. Manual Mode)	9.0 (6.9-11.1)	**<.001**
	Previous therapy (CSII vs. MDI)		0.6 (-3.2-4.5)	0.746
SG CV (%)	Period	Automode 3M (vs. Manual Mode)	-0.9 (-2.4-0.5)	0.202
	Period	Automode 6M (vs. Manual Mode)	-0.2 (-1.4-1.0)	0.745
	Previous therapy (CSII vs. MDI)		1.5 (-0.4-3.3)	0.113
GMI (%)	Period	Automode 3M (vs. Manual Mode)	-0.3 (-0.5--0.2)	**<.001**
	Period	Automode 6M (vs. Manual Mode)	-0.4 (-0.5--0.3)	**<.001**
	Previous therapy (CSII vs. MDI)		-0.1 (-0.2-0.1)	0.442
Basal volume (%)	Period	Automode 3M (vs. Manual Mode)	-5.3 (-7.8--2.8)	**<.001**
	Period	Automode 6M (vs. Manual Mode)	-4.3 (-6.4--2.2)	**<.001**
	Previous therapy (CSII vs. MDI)*		3.8 (0.9-6.6)	**0.010**
Bolus volume (%)	Period	Automode 3M (vs. Manual Mode)	4.8 (2.3-7.4)	**<.001**
	Period	Automode 6M (vs. Manual Mode)	4.3 (2.1-6.5)	**<.001**
	Previous therapy (CSII vs. MDI)		-3.9 (-6.8--1.1)	**0.008**
TDD/kg (units)	Period	Automode 3M (vs. Manual Mode)	0.1 (0.0-0.1)	**0.008**
	Period	Automode 6M (vs. Manual Mode)	0.1 (0.0-0.1)	**<.001**
	Previous therapy (CSII vs. MDI)		0.1 (-0.0-0.2)	0.167
CHO/kg (gr)	Period	Automode 3M (vs. Manual Mode)	0.1 (-0.4-0.5)	0.810
	Period	Automode 6M (vs. Manual Mode)	0.1 (-0.3-0.4)	0.665
	Previous therapy (CSII vs. MDI)		-0.3 (-1.2-0.6)	0.483
BMI (kg/m²)	Period	Automode 3M (vs. Manual Mode)	0.0 (-0.2-0.3)	0.776
	Period	Automode 6M (vs. Manual Mode)	0.1 (-0.1-0.3)	0.205
	Previous therapy (CSII vs. MDI)		0.4 (-1.0-1.9)	0.536
BMI z score	Period	Automode 3M (vs. Manual Mode)	-0.1 (-0.2-0.0)	0.153
	Period	Automode 6M (vs. Manual Mode)	-0.1 (-0.2-0.0)	**0.050**
	Previous therapy (CSII vs. MDI)		-0.1 (-0.5-0.3)	0.567

*interaction test is significant

Abbreviations: Automode 3M, first 14 days after 3 months; Automode 6M, first 14 days after 6 months; BMI, body mass index; CI, confidence interval; CSII, continuous subcutaneous insulin infusion; GMI, glucose management indicator; MDI, multiple daily injections; SG CV, sensor glucose coefficient of variation; TDD, total daily dose; TIR, time in range. Bold indicates statistically significant.
